# Diagnostic Value of Inflammatory Factors in Pathology of Bladder Cancer Patients

**DOI:** 10.3389/fmolb.2020.575483

**Published:** 2020-11-05

**Authors:** Xingxing Tang, Yudong Cao, Jia Liu, Shuo Wang, Yong Yang, Peng Du

**Affiliations:** Key Laboratory of Carcinogenesis and Translational Research (Ministry of Education), Department of Urology, Peking University Cancer Hospital & Institute, Beijing, China

**Keywords:** inflammatory factors, bladder cancer, neutrophil to lymphocyte ratio, derived neutrophil to lymphocyte ratio, platelet to lymphocyte ratio, lymphocyte to monocyte ratio, systemic immune-inflammation index, prognostic nutritional index

## Abstract

We conducted this study to evaluate the diagnostic value of Inflammatory Factors (IFs) in the pathology of bladder cancer patients. The patients who were diagnosed with urothelial bladder carcinoma (bladder cancer) and underwent surgical treatment in our center from 2014 to 2019 were enrolled. The values of Neutrophil to Lymphocyte Ratio (NLR), derived Neutrophil to Lymphocyte Ratio (dNLR), Platelet to Lymphocyte Ratio (PLR), Lymphocyte to Monocyte Ratio (LMR), Systemic Immune-inflammation Index (SII), and Prognostic Nutritional Index (PNI) were calculated by blood routine test results before operation. After obtaining the postoperative pathology of the patients, the Area Under Curve (AUC) of Receiver Operating Characteristic (ROC) curves was calculated to evaluate the diagnostic value of these IFs in pathology and their corresponding cut-off values. A total of 641 bladder cancer patients were enrolled. The median values of NLR, dNLR, PLR, LMR, SII, and PNI were 6.33, 4.09, 156.47, 2.66, 1114.29, and 51.45, respectively. Grouped patients according to the pathological grade, the NLR, dNLR, PLR, and SII of the high-grade group were significantly higher than those of the low-grade group (*P* < 0.001, *P* < 0.001, *P* < 0.001, and *P* < 0.001, respectively), while the LMR and PNI were significantly lower than those of the low-grade group (*P* = 0.003 and *P* < 0.001). Divided patients into non-muscle invasion group (Tis + Ta + T1) and muscle invasion group (T2 + T3 + T4), in which NLR, dNLR, PLR, and SII in the muscle invasion group were significantly higher than those in the non-muscle invasion group (*P* < 0.001, *P* < 0.001, *P* < 0.001, and *P* < 0.001, respectively), while LMR and PNI were significantly lower than those in the low-grade group (*P* = 0.012 and *P* < 0.001). ROC curves analyses showed that NLR, dNLR, PLR, LMR, SII, and PNI had predictive value for pathological grade (*P* < 0.001, *P* < 0.001, *P* < 0.001, *P* < 0.001, *P* < 0.001, *P* < 0.001, and *P* < 0.001, respectively) and muscle invasion (*P* < 0.001, *P* < 0.001, *P* < 0.001, *P* < 0.001, *P* < 0.001, *P* < 0.001, and *P* < 0.001, respectively). The results suggest the higher NLR, dNLR, PLR, SII, and lower LMR and PNI are associated with higher risk of high-grade and muscle invasive disease. However, this conclusion needs to be further clarified in the future.

## Introduction

Blood sampling test is a routine examination for all admitted patients. White blood cell count, neutrophil count, monocyte count, lymphocyte count, platelet count and albumin concentration are common indicators in blood sampling test. Some inflammatory factors (IFs) can be easily obtained by simple calculation of these indicators. Commonly used IFs include Neutrophil to Lymphocyte Ratio (NLR), derived Neutrophil to Lymphocyte Ratio (dNLR), Platelet to Lymphocyte Ratio (PLR), Lymphocyte to Monocyte Ratio (LMR), and Systemic Immune-inflammation Index (SII) (Luo et al., 2018; Rajwa et al., 2018; Zhang et al., 2019). Currently, relevant studies have confirmed that these IFs have certain diagnostic value in different tumors (Rossi et al., 2017; Urabe et al., 2018; Losada et al., 2019). In addition, there is another IF named Prognostic Nutritional Index (PNI), which takes into account both the inflammation level and nutritional status of patients. In recent years, several studies have found that PNI has predictive and prognostic value in urothelial bladder carcinoma (bladder cancer) (Peng et al., 2017; Karsiyakali et al., 2020), which is a common tumor in urology (Siegel et al., 2017). The pathological results have a great impact on the choice of treatment and prognosis in bladder cancer patients. Therefore, exploring the examination with diagnostic value for bladder cancer pathology is of important clinical significance. At present, a number of studies have confirmed that IFs have certain diagnostic value for bladder cancer pathology (Lee et al., 2015; Rajwa et al., 2018), and our previous studies also found that NLR is associated with the risk of high-grade disease (Tang et al., 2017a,b). Considering these studies only involved some of the above IFs, we carried out this study including all these IFs to analysis their diagnostic value in the pathology of bladder cancer patients.

## Materials and Methods

### Study Subjects

This single-center retrospective study included all patients diagnosed with bladder cancer in the outpatient clinic of Peking University Cancer Hospital from 2014 to 2019 and admitted for surgical treatment, including transurethral resection of bladder tumor (TURBT), partial cystectomy and total cystectomy. All enrolled patients were required to have no chemoradiotherapy before surgery and diagnosed with urothelial bladder carcinoma pathologically. Patients with other chronic inflammatory diseases or immune system diseases were excluded.

### Study Methods

The basic information of patients (hospitalization number, age, gender), pathological data (tumor grade, tumor TNM stage), blood test results (white blood cell count, neutrophil count, monocyte count, lymphocyte count, platelet count, albumin content) were collected through the hospital’s medical record management system. Pathological staging and histological grading of bladder cancer were based on the American Joint Commission TNM staging system on Bladder Cancer (seventh edition, 2010) (Edge and Compton, 2010). All blood sampling results were completed by the Laboratory Department of Peking University Cancer Hospital, which were the results of the first fasting blood sampling after the patient was admitted to the hospital. All pathology reports were interpreted by urologists in the Department of Pathology, Peking University Cancer Hospital. NLR was calculated as neutrophil count (×10^9^/L)/lymphocyte count (×10^9^/L), dNLR was calculated as neutrophil count (×10^9^/L)/(leukocyte count (×10^9^/L) – neutrophil count (×10^9^/L)), PLR was calculated as platelet count (×10^9^/L)/lymphocyte count (×10^9^/L), LMR was calculated as lymphocyte count (×10^9^/L)/monocyte count (×10^9^/L), SII was calculated as platelet count (×10^9^/L) × neutrophil count (×10^9^/L)/lymphocyte count (×10^9^/L), and PNI was measured as albumin (g/L) + 5 × lymphocyte count (×10^9^/L) (Luo et al., 2018; Rajwa et al., 2018; Zhang et al., 2019).

### Statistical Analyses

Patients’ characteristics were summarized using descriptive statistics, with categorical variables expressed as numbers and percentages and continuous variables as medians. *T*-test was used to compare continuous variables with normal distribution between groups, and chi-square test and cross-table test were used to compare categorical variables between groups. Receiver Operating Characteristic (ROC) curves were plotted to calculate the Area Under Curve (AUC) of IFs, and Youden index were calculated to select the best cut-off values. The plotting of ROC curves and the calculation of the optimal cut-off value were completed using MedCalc software (version 19), and the rest of the statistics were completed using Stata software (version 15). All tests were two-sided and *P* < 0.05 was considered statistically significant.

## Results

### Patients’ Characteristics

This study eventually included 641 eligible patients with bladder cancer, with a median age of 67 years. 501 (78.16%) patients were male patients, and more than half of the patients (52.57%) had a history of smoking. In terms of pathological results, the number of low-grade and high-grade patients was 227 and 414, respectively. Most of the patients were diagnosed with Ta (57.57%) and T1 (25.43%) tumors, the other 15.91% of them were diagnosed with muscle invasive disease. The median values of NLR, dNLR, PLR, LMR, SII, and PNI were 6.33, 4.09, 156.47, 2.66, 1114.29, and 51.45, respectively. The patients’ characteristics are shown in Table 1.

**TABLE 1:** Patients’ characteristics.

**Characteristics**	**Total**
Patients, n	641
Age, year, median (IQR)	67 (60–75)
Sex, n (%)	
Male	501 (78.16)
Female	140 (21.84)
Smoke, n (%)	
No	304 (47.43)
Yes	337 (52.57)
Pathological grade, n (%)	
Low grade	227 (35.41)
High grade	414 (64.59)
Pathological T-stage, n (%)	
Tis	7 (1.09)
Ta	369 (57.57)
T1	163 (25.43)
T2	59 (9.20)
T3	34 (5.30)
T4	9 (1.40)
Muscle invasion, n (%)	
No (Tis + Ta + T1)	539 (84.09)
Yes (T2 + T3 + T4)	102 (15.91)
Leukocyte count, ×10^9^/L, median (IQR)	9.26 (6.88–12.34)
Neutrophil count, ×10^9^/L, median (IQR)	7.26 (4.87–10.53)
Lymphocyte count, ×10^9^/L, median (IQR)	1.24 (0.87–1.70)
Platelet count, ×10^9^/L, median (IQR)	198 (162–243)
Monocyte count, ×10^9^/L, median (IQR)	0.49 (0.35–0.66)
Albumin, g/L, median (IQR)	45.50 (42.80–47.50)
NLR, median (IQR)	6.33 (3.14–10.94)
dNLR, median (IQR)	4.09 (2.25–6.79)
PLR, median (IQR)	156.47 (120.14–225.20)
LMR, median (IQR)	2.66 (1.63–3.94)
SII, median (IQR)	1114.29 (626.15–2124.56)
PNI, median (IQR)	51.45 (48.45–54.9)

*NLR, Neutrophil to Lymphocyte Ratio; dNLR, derived Neutrophil to Lymphocyte Ratio; PLR, Platelet to Lymphocyte Ratio; LMR, Lymphocyte to Monocyte Ratio; SII, Systemic Immune-inflammation Index; PNI, Prognostic Nutritional Index; IQR, Interquartile Range.*

### Comparison of the Value of IFs Between Different Groups

Grouped patients according to the age, gender, smoking history, pathological grade, muscle invasion, and compared whether there were significant differences between the two groups in these IFs. Grouped by age, the patients were divided into high group (≥60) and low group (<60). The dNLR of low group was significantly higher than that of high group (*P* = 0.018), while the PNI of high group was significantly higher than that of low group (*P* < 0.001). There were no significant differences in NLR, PLR, LMR, and SII between the two groups. In terms of gender, LMR in female patients was significantly higher than that in male patients (*P* < 0.001), and there was no significant difference in other IFs between the two groups. The PLR of smoking group was significantly lower than that of non-smoking group (*P* = 0.043), while the PNI was significantly higher than that of non-smoking group (*P* = 0.001). Grouped according to the pathological grade, the results showed that all IFs had significant differences between the two groups, in which the NLR, dNLR, PLR, and SII in the high-grade group were significantly higher than those in the low-grade group (*P* < 0.001, *P* < 0.001, *P* < 0.001 and *P* < 0.001, respectively), while the LMR and PNI were significantly lower than those in the low-grade group (*P* = 0.003 and *P* < 0.001). According to whether the tumors invaded the muscle or not, the patients were divided into non-muscle invasion group (Tis + Ta + T1) and muscle invasion group (T2 + T3 + T4). There were significant differences in all IFs between the two groups, among which NLR, dNLR, PLR, and SII in the muscle invasion group were significantly higher than those in the non-muscle invasion group (*P* < 0.001, *P* < 0.001, *P* < 0.001 and *P* < 0.001, respectively), while LMR and PNI were significantly lower than those in the low-grade group (*P* = 0.012 and *P* < 0.001). The comparison of the values of IFs between different groups is shown in Table 2.

**TABLE 2:** Comparison of the values of inflammatory factors between different groups.

**Variables**	**N**	**NLR**			***P*- value**	**dNLR**			***P*- value**	**PLR**			***P*- value**	**LMR**			***P*- value**	**SII**			***P*- value**	**PNI**			***P*- value**
		**Mean**	**95% CI**			**Mean**	**95% CI**			**Mean**	**95% CI**			**Mean**	**95% CI**			**Mean**	**95% CI**			**Mean**	**95% CI**		
			**LL**	**UL**			**LL**	**UL**			**LL**	**UL**			**LL**	**UL**			**LL**	**UL**			**LL**	**UL**	
Age, year					0.065				0.018				0.557				0.478				0.450				<0.001
≥60	159	7.47	6.27	8.67		4.44	3.94	4.94		192.88	170.84	214.91		3.63	3.20	4.06		1691.67	1400.67	1982.67		53.84	53.06	54.62	
<60	482	9.20	8.22	10.19		5.34	4.95	5.74		201.21	186.96	215.46		3.31	2.81	3.80		1870.03	1621.40	2118.65		50.85	50.39	51.30	
Gender					0.087				0.175				0.579				<0.001				0.385				0.254
Female	140	7.46	6.12	8.80		4.70	4.00	5.39		205.57	182.30	228.84		4.99	3.42	6.55		1658.12	1346.72	1969.51		51.14	50.18	52.11	
Male	501	9.14	8.19	10.09		5.24	4.87	5.60		197.34	183.39	211.29		2.94	2.72	3.16		1872.64	1631.50	2113.78		51.71	51.27	52.16	
Smoke					0.756				0.819				0.043				0.050				0.254				0.001
No	304	8.91	7.56	10.25		5.16	4.64	5.68		212.15	192.25	232.05		3.79	3.03	4.55		1948.24	1583.13	2313.35		50.90	50.29	51.51	
Yes	337	8.65	7.73	9.57		5.08	4.68	5.49		187.40	173.31	201.50		3.02	2.76	3.28		1715.32	1523.08	1907.57		52.21	51.68	52.74	
Pathological grade					<0.001				<0.001				<0.001				0.003				<0.001				<0.001
Low grade	227	6.08	5.41	6.74		3.94	3.60	4.29		167.11	156.22	177.99		4.16	3.19	5.12		1249.80	1118.22	1381.39		53.46	52.85	54.07	
High grade	414	10.25	9.09	11.41		5.76	5.31	6.22		216.71	199.30	234.11		2.96	2.69	3.23		2141.60	1844.12	2439.08		50.56	50.06	51.07	
Muscle invasion					<0.001				<0.001				<0.001				0.012				<0.001				<0.001
No (Tis + Ta + T1)	539	7.77	6.91	8.64		4.65	4.31	5.00		185.78	173.33	198.23		3.60	3.16	4.04		1578.21	1363.98	1792.44		52.10	51.67	52.53	
Yes (T2 + T3 + T4)	102	14.06	12.30	15.81		7.58	6.79	8.36		269.76	235.43	304.08		2.26	1.70	2.81		3134.04	2653.47	3614.61		48.88	47.90	49.86	

*NLR, Neutrophil to Lymphocyte Ratio; dNLR, derived Neutrophil to Lymphocyte Ratio; PLR, Platelet to Lymphocyte Ratio; LMR, Lymphocyte to Monocyte Ratio; SII, Systemic Immune-inflammation Index; PNI, Prognostic Nutritional Index; 95% CI, 95% Confidence Interval; LL, Lower Limit; UL, Upper Limit.*

### Diagnostic Value of IFs in Pathology of Patients With Bladder Cancer

**FIGURE 1:**
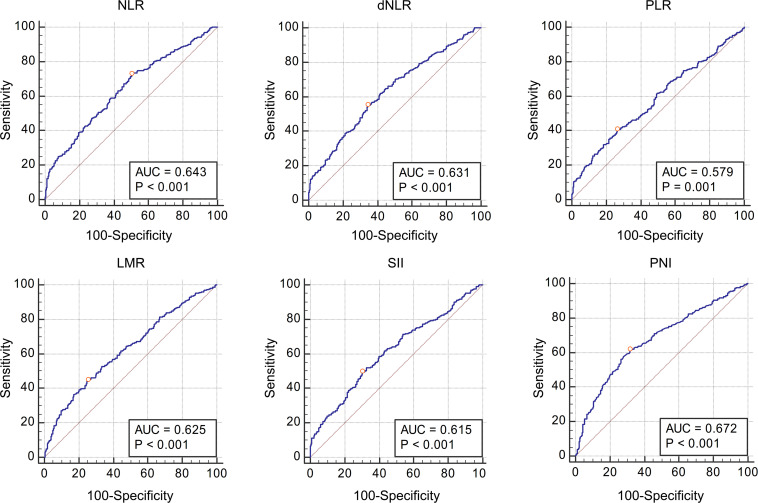
The Receiver Operating Characteristic curves and Area Under Curve of NLR, dNLR, PLR, LMR, SII, and PNI for pathological grade. All these inflammatory factors had predictive value for pathological grade (*P* < 0.001, *P* < 0.001, *P* < 0.001, *P* < 0.001, *P* < 0.001 and *P* < 0.001 respectively), and the optimal cut-off values were 4.24, 4.23, 190.44, 2.11, 1337.80, and 51.70, respectively.

**FIGURE 2:**
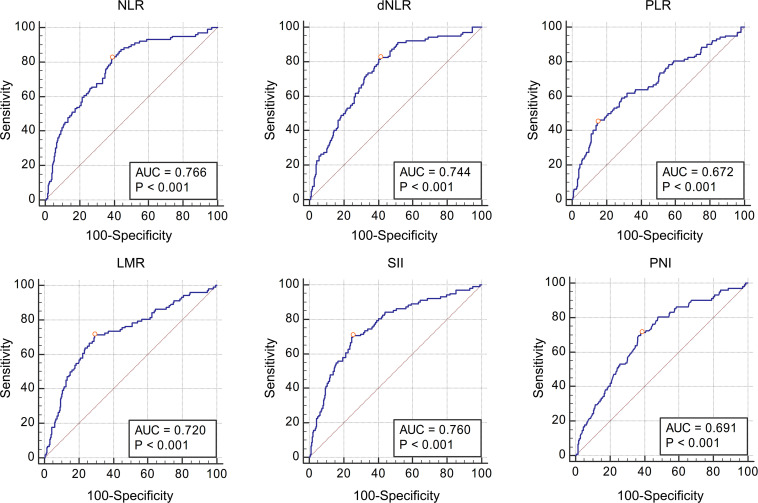
The Receiver Operating Characteristic curves and Area Under Curve of NLR, dNLR, PLR, LMR, SII, and PNI for muscle invasion. All these inflammatory factors had predictive value for pathological grade (*P* < 0.001, *P* < 0.001, *P* < 0.001, *P* < 0.001, *P* < 0.001, and *P* < 0.001 respectively), and the optimal cut-off values were 6.85, 4.23, 248.86, 2.01, 1723.14, and 50.90, respectively.

Table 3 shows the AUC and cut-off values of the IFs for pathological grade and muscle invasion. ROC curve analyses showed that NLR, dNLR, PLR, LMR, SII, and PNI had predictive value for pathological grade (high-grade vs. low-grade) (*P* < 0.001, *P* < 0.001, *P* < 0.001, *P* < 0.001, *P* < 0.001, *P* < 0.001, and *P* < 0.001, respectively), and their optimal cut-off values were 4.24, 4.23, 190.44, 2.11, 1337.80, and 51.70, respectively, while the corresponding AUC were 0.643, 0.631, 0.579, 0.625, 0.615, and 0.672, respectively (Figure 1). NLR, dNLR, PLR, LMR, SII, and PNI all had predictive value for muscle invasion (muscle invasion vs. non-muscle invasion) (*P* < 0.001, *P* < 0.001, *P* < 0.001, *P* < 0.001, *P* < 0.001, *P* < 0.001, and *P* < 0.001, respectively), and their optimal cut-off values were 6.85, 4.23, 248.86, 2.01, 1723.14, and 50.90, with corresponding AUC of 0.766, 0.744, 0.672, 0.720, 0.760, and 0.691, respectively (Figure 2).

**TABLE 3:** The Area Under Curve and cut-off values of the inflammatory factors for pathological grade and muscle invasion.

	**Cut-off value**	**Sensitivity**	**Specificity**	**AUC**	**SE**	**95% CI**		***Z-*value**	***P*-value**
						**LL**	**UL**		
**Pathological grade**									
NLR	4.24	72.71	49.78	0.643	0.022	0.604	0.680	6.423	<0.001
dNLR	4.23	55.31	65.64	0.631	0.023	0.592	0.668	5.808	<0.001
PLR	190.44	40.58	73.57	0.579	0.023	0.540	0.618	3.456	<0.001
LMR	2.11	44.93	74.45	0.625	0.023	0.587	0.663	5.568	<0.001
SII	1337.80	49.76	69.60	0.615	0.023	0.576	0.653	5.095	<0.001
PNI	51.70	61.59	68.28	0.672	0.022	0.634	0.708	7.915	<0.001
**Muscle invasion**									
NLR	6.85	82.35	61.04	0.766	0.025	0.731	0.798	10.525	<0.001
dNLR	4.23	82.35	58.81	0.744	0.025	0.708	0.777	9.619	<0.001
PLR	248.86	45.10	84.97	0.672	0.031	0.634	0.709	5.511	<0.001
LMR	2.01	71.57	70.87	0.720	0.029	0.684	0.755	7.515	<0.001
SII	1723.14	70.59	74.58	0.760	0.027	0.725	0.792	9.747	<0.001
PNI	50.90	71.57	61.41	0.691	0.028	0.653	0.726	6.805	<0.001

*NLR, Neutrophil to Lymphocyte Ratio; dNLR, derived Neutrophil to Lymphocyte Ratio; PLR, Platelet to Lymphocyte Ratio; LMR, Lymphocyte to Monocyte Ratio; SII, Systemic Immune-inflammation Index; PNI, Prognostic Nutritional Index; AUC, Area Under Curve; 95% CI, 95% Confidence Interval; SE, Standard Error; LL, Lower Limit; UL, Upper Limit.*

## Discussion

Blood sampling test is a routine admission examination for each patient. By obtaining the white blood cell count, neutrophil count, monocyte count, lymphocyte count, platelet count and albumin concentration, it is convenient to calculate the values of NLR, dNLR, PLR, LMR, SII, and PNI. Therefore, the acquisition of the aforementioned IFs has no cost, and there is no need for patients to bear additional injuries at all. For this reason, more and more studies have begun to explore the diagnostic and prognostic value of these IFs in different tumors, and at present, meaningful results have been obtained in gastric cancer, colon cancer and breast cancer, etc. (Rossi et al., 2017; Urabe et al., 2018; Losada et al., 2019). Nowadays there is still no clear mechanism as to why these IFs can predict the tumor progression. Studies have suggested that leukocytes are indicators of systemic inflammation. When patients have infection or inflammation, leukocytes usually rise. In addition, cancer cells can also produce colony-stimulating factors to promote the increase of leukocyte counts, so the increase of leukocytes is related to tumor progression (Tachibana et al., 1995). Neutrophils may promote tumor neocapillary by releasing elastase and destroying extracellular matrix, and in turn promote tumor progression (Ginzberg et al., 2001). Monocytes can increase the number of tumor-associated macrophages in the blood, which is thought to be associated with tumor progression (Galdiero et al., 2013). Lymphocytes are related to the body’s immunity, when the number of lymphocytes in tumor tissues decreases, local immunity decreases, which in turn provides an immunocompromised environment for tumor growth (Lee et al., 2012). Platelets could wrap around the tumor cells entering the circulatory system, reduce their immunogenicity, help the tumor cells to escape from immune surveillance and immune clearance, and then help the spread of tumor (Schlesinger, 2018). Albumin is the body’s nutritional indicators, and low albumin usually indicates the body’s malnutrition. PNI includes lymphocyte count and albumin concentration, therefore it is an index that comprehensively reflects the body’s immunity and nutritional level (Karsiyakali et al., 2020).

Bladder cancer is one of the most common tumors in urology (Siegel et al., 2017), and the depth of tumor invasion will directly affect the treatment and prognosis of patients. Usually this needs to be clarified by imaging and cystoscopy, but micro muscle invasion cannot be observed by imaging, while cystoscopy is usually limited to the muscle layer and is not help for deeper invasion. Therefore, it is of great clinical significance to explore more examinations with diagnostic value in this regard. Although IFs can’t replace the role of imaging and cystoscopy in the diagnosis of bladder cancer, considering IFs are simple and easy to obtain, they are meaningful even if they can only provide limited diagnostic information. Relevant studies have shown that IFs have diagnostic value for the pathology of bladder cancer (Rajwa et al., 2018; Kumarasamy et al., 2019), and our previous studies have also found that high NLR is associated with high-grade disease (Tang et al., 2017a,b). This study was further expanded by including all commonly used IFs in the analysis.

The patients were grouped according to pathological grade and muscle invasion, and the values of IFs in different groups were directly compared. The results showed that NLR, dNLR, PLR, and SII in the high-grade group were significantly higher than those in the low-grade group, while LMR and PNI were significantly lower than those in the low-grade group, suggesting that NLR, dNLR, PLR, and SII were positively correlated with pathological grade, while LMR and PNI were negatively correlated. This is consistent with the previous analysis, that is, the increase of leukocytes, neutrophils, platelets, monocytes is conducive to the growth and progression of tumor cells, while the increase of lymphocytes and albumin means stronger immunity and better nutritional status, is not conducive to the growth and progression of tumor cells, so the higher the LMR and PNI, the more unfavorable to the progression of tumor. The same phenomenon was also observed in the muscle invasion group, where NLR, dNLR, PLR, and SII were significantly higher in the muscle invasion group than in the non-muscle invasion group, while LMR and PNI were significantly lower than in the non-muscle invasion group. On this basis, we further analyzed the diagnostic value of IFs in bladder cancer pathology, calculated AUC by plotting ROC curves, and selected the best cut-off values. We found that NLR, dNLR, PLR, LMR, SII, and PNI had diagnostic value for pathological grade and muscle invasion. According to the definition of relevant literatures, the diagnostic value of AUC value between 0.7 and 0.8 is acceptable, AUC value between 0.6 and 0.7 is poor, and AUC value between 0.5 and 0.6 indicates no diagnostic value (Mandrekar, 2010). Although NLR, dNLR, PLR, LMR, SII, and PNI have diagnostic value for the pathological grade of bladder cancer, all the values of AUC are between 0.6 and 0.7, suggesting these IFs only have limited diagnostic value for the pathological grade. In terms of muscle invasion, the AUC values of NLR, dNLR, LMR, and SII ranged from 0.7 to 0.8, suggesting that these four IFs have acceptable diagnostic value for muscle invasion, while the AUC of PLR and PNI ranged from 0.6 to 0.7, suggesting limited diagnostic value. Combined with the previous analysis, it can be concluded that the higher the NLR, dNLR, PLR, and SII, the lower the LMR and PNI, the higher the risk of high-grade and muscle invasive disease. It should be noted that for different diagnoses, the cut-off values of above IFs are different, such as NLR has diagnostic value for both pathological grade and muscle invasion, but the cut-off values are 4.24 and 6.85, respectively. Therefore, follow-up studies with large sample sizes are needed to clarify the cut-off values of these IFs. At present, the cut-off values of these IFs in different studies are different, and clinical application of these IFs is still difficult.

In fact, several studies have similarities with our research (Cantiello et al., 2018; Rajwa et al., 2018; Ninomiya et al., 2020), but compared with these studies, our research still has many differences. The main differences between the first study (Rajwa et al., 2018) and our study are as follows. First of all, the subjects were different. That study only included patients who underwent radical cystectomy, while our study covered all patients with bladder cancer, including those who underwent TURBT, partial cystectomy and radical cystectomy. Secondly, our study is the first to analyze the diagnostic value of SII and PNI in patients with bladder cancer, which is not involved in that study. Third, due to the different population, our sample size is significantly larger. We believe that a larger sample size can provide more reliable conclusions. The second study (Cantiello et al., 2018) was the opposite of the first. Only patients with non-muscle invasive bladder cancer were included, and those with muscle invasion undergoing radical cystectomy were not included. The IFs analyzed in this study included NLR, PLR and LMR, but not dNLR, SII, and PNI. The third study (Ninomiya et al., 2020) was similar to the first study. The subjects were patients undergoing radical cystectomy. The IFs analyzed included NLR, LPR (PLR), MLR (LMR), and PNI, excluding dNLR and SII. In general, we think that compared with the above studies, the biggest difference in our study is the analysis of more IFs, especially for SII and PNI, there are very few related studies in the field of bladder cancer. In addition, we also included more patients. It is also important that the above three studies focus on the predictive value of the IFs in patients’ survival, while our research focuses on the diagnostic value of the IFs for the severity of disease (pathological staging, whether tumor invades bladder muscle). The purpose of our study is to provide relevant evidence to help clinicians make a preliminary assessment of the patient’s condition when they see the blood routine test results of patients considering the diagnosis of bladder cancer. In this way, when making the next diagnosis and treatment plan, it could be more targeted.

It is worth mentioning that Bladder Tumor Antigen (BTA) and Nuclear Matrix Protein 22 (NMP22) are also non-invasive tests for bladder cancer. Relevant studies have confirmed that these two tests have certain value in the diagnosis of bladder cancer (Zippe et al., 1999; Giannopoulos et al., 2001). If IFs, BTA and NMP tests are combined, the diagnostic accuracy of bladder cancer might be improved. However, although the diagnostic value of IFs in bladder cancer might be not comparable to BTA and NMP22, considering that in China and most parts of the world, most hospitals do not carry out these two tests and other similar tests due to the need for certain costs and specific equipment, the biggest advantage of IFs is low cost and easy to popularize.

One of the shortcomings of this study is its single center design. Although the sample size is enough, this might bring some bias. But on the other hand, the single center design also brings some advantages. All operations were performed by the urology department of Peking University Cancer Hospital. Blood tests and pathology were performed by the urology group of the Laboratory Department and Pathology Department of Peking University Cancer Hospital. Therefore, the quality of operation, blood tests, and pathological results are consistent, and we believe single center research also has its advantages. Another limitation of this study is that most of the included patients were non-muscle invasive patients. Considering the large prognostic gap between muscle invasive and non-muscle invasive disease, we did not analyze the prognostic value of IFs for survival, and we would conduct a separate analysis for muscle invasive bladder cancer patients after the inclusion of more patients. For the same reason, since most patients underwent TURBT and there was no information about lymph node metastasis in the pathology, we did not analyze the diagnostic value of IFs in predicting lymph node metastasis in this study, which will be analyzed separately after more patients with total cystectomy are included in the future.

## Conclusion

Our study shows that NLR, dNLR, PLR, LMR, SII, and PNI are of diagnostic value for pathological grade and muscle invasion in patients with bladder cancer. The higher NLR, dNLR, PLR, SII, and lower LMR and PNI are associated with higher risk of high-grade and muscle invasive disease. However, this conclusion and the optimal cut-off values need to be further clarified by larger studies in the future.

## Data Availability Statement

The raw data supporting the conclusions of this article will be made available by the authors, without undue reservation.

## Ethics Statement

The studies involving human participants were reviewed and approved by the Peking University Cancer Hospital. Written informed consent for participation was not required for this study in accordance with the national legislation and the institutional requirements. Written informed consent was not obtained from the individual(s) for the publication of any potentially identifiable images or data included in this article.

## Author Contributions

XT contributed to prepare the manuscript and the statistical analysis. PD put forward the concept of the study and designed the study. YY reviewed the manuscript. YC, SW, and JL contributed to the data acquisition, analysis, and interpretation. XT carried out the data analysis. All authors read and approved the final manuscript.

## Conflict of Interest

The authors declare that the research was conducted in the absence of any commercial or financial relationships that could be construed as a potential conflict of interest.
